# Off-target effects in CRISPR-Cas genome editing for human therapeutics: Progress and challenges

**DOI:** 10.1016/j.omtn.2025.102636

**Published:** 2025-07-17

**Authors:** Nechama Kalter, Carla Fuster-García, Alfredo Silva, Víctor Ronco-Díaz, Stefano Roncelli, Giandomenico Turchiano, Jan Gorodkin, Toni Cathomen, Karim Benabdellah, Ciaran Lee, Ayal Hendel

**Affiliations:** 1 The Institute for Advanced Materials and Nanotechnology, The Mina and Everard Goodman Faculty of Life Sciences, Bar-Ilan University, Ramat-Gan 5290002, Israel; 2 Institute for Transfusion Medicine and Gene Therapy, Medical Center–University of Freiburg, 79106 Freiburg, Germany; 3 Center for Chronic Immunodeficiency (CCI), Faculty of Medicine, University of Freiburg, 79106 Freiburg, Germany; 4 San Raffaele Telethon Institute for Gene Therapy, Novel Gene Therapy Strategies Unit, 20132 Milan, Italy; 5 Department of Genomic Medicine, Pfizer-University of Granada-Andalusian Regional Government Centre for Genomics and Oncological Research (GENYO), 18016 Granada, Spain; 6 Center for Non-coding RNA in Technology and Health, Department of Veterinary and Animal Sciences, University of Copenhagen, 1870 Frederiksberg, Denmark; 7 Infection, Immunity, and Inflammation Research and Teaching Department, Great Ormond Street Institute of Child Health, University College London, London WC1N 1EH, UK; 8 Cell and Gene Therapy Safety, Clinical Pharmacology and Safety Sciences R&D, AstraZeneca, Cambridge CB2 0AA, UK; 9 School of Biochemistry and Cell Biology, University College Cork, T12 YN60 Cork, Ireland

**Keywords:** MT: Clinical Applications, CRISPR-Cas9, off-target, safety, genome editing, gene therapy

## Abstract

Targeted nucleases, primarily CRISPR-Cas-based systems, have revolutionized genome editing by enabling precise modification of target genes or transcripts. Many pre-clinical and clinical studies leverage this technology to develop treatments for human diseases; however, substantial off-target genotoxicity concerns delay its clinical translation. Despite the development of a wide array of tools, assays, and technologies aimed at identifying and quantifying off-target effects, the absence of standardized guidelines leads to inconsistent practices across studies. This review highlights the key challenges and potential solutions in ensuring the safety of gene editing studies for therapeutic applications, focusing on gRNA design, off-target sites prediction, and off-target activity measurement.

## Graphical abstract

**Figure:**
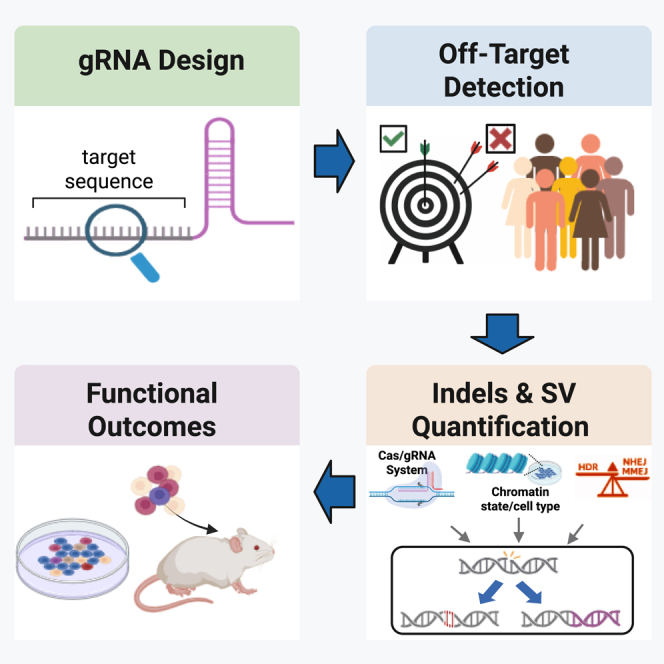

## Teaser

Kalter and colleagues provide a comprehensive review of the key steps in designing a safe and precise guide RNA for CRISPR-Cas therapeutic applications, focusing on guide RNA design, the detection and quantification of off-target activity, the identification of structural variations, and the functional assessment of potential genotoxicity.

## Introduction

### The CRISPR-Cas toolkit

**Figure 1:**
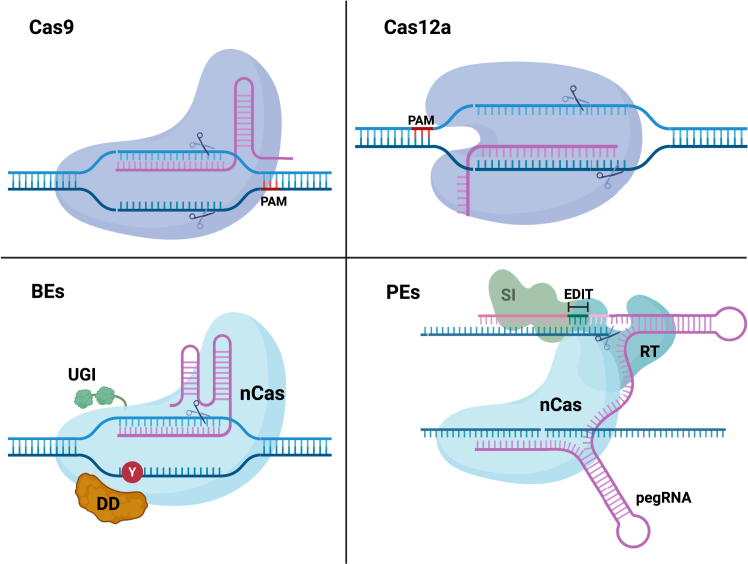
CRISPR-Cas DNA editors General structure of Cas9 and Cas12a nucleases, BEs and PEs, either with or without serine integrase domain fusion. DD, deaminase domain; nCas, nicking Cas; PAM, protospacer adjacent motif; RT, reverse transcriptase; SI, serine integrase; UGI, uracyl-glycosilase inhibitor; Y, A-or-C bases. Created with BioRender.com.

**Figure 2:**
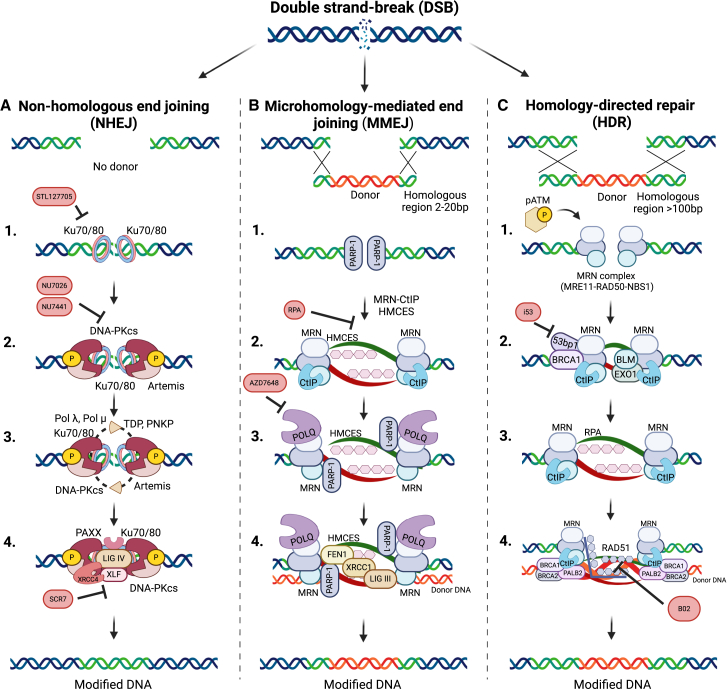
DSB repair pathways and small-molecule inhibitors The repair of DSBs follows three major pathways: HDR, NHEJ, MMEJ. (A) In the HDR pathway, MRN and CtIP initiate DNA end resection; RAD51 then replaces RPA on single-stranded DNA, enabling homology-based strand invasion and template-directed repair. HDR inhibitors include AICAR and B02, targeting RAD52 and RAD51, respectively. (B) In the NHEJ repair pathway, Ku70/Ku80 binds to DNA ends, DNA-protein kinase catalytic subunit promotes end processing if needed, and XRCC4-DNA ligase IV complex seals the DNA break. (C) In the MMEJ pathway, PARP-1 competes with Ku70/80 for DSB binding, MRN/CtIP mediates limited resection, and POLQ aligns microhomologous sequences, facilitating error-prone synthesis and repair by the LIG3/XRCC1 complex. Created with BioRender.com.

Clustered regularly interspaced short palindromic repeats (CRISPR)-Cas systems have been rapidly adopted in the genome-editing field, with promising applications in treating human diseases. Originally evolved as an adaptive immune mechanism in bacteria and archaea to combat bacteriophage infection, the CRISPR-Cas technology enables precise, base pair resolution modification at nearly any genomic locus (Figure 1).^1^^,^^2^ The CRISPR-Cas system includes two main components: an endonuclease from the Cas family and a guide RNA (gRNA). The diverse Cas family includes nucleases of varying sizes and cutting patterns; among them, Cas9 and Cas12a have already been tested in clinical trials.^3^ The gRNA includes a 20- to 24-nt spacer sequence that directs the endonuclease into a complementary sequence in the genome. Upon reaching the DNA target site, the Cas nuclease induces a double-strand break (DSB), subsequently repaired by the cell’s intrinsic repair mechanisms: predominantly non-homologous end joining (NHEJ) or homology-directed repair (HDR).^4^^,^^5^^,^^6^ NHEJ is a rapid, error-prone process that involves a simple ligation of the two ends of the break, often introducing short insertion or deletions (indels) at the DNA cut site, making it ideal for generating frameshift or nonsense mutations.^7^^,^^8^^,^^9^ A less common repair pathway is alternative NHEJ (alt-NHEJ), also referred to as microhomology-mediated end joining (MMEJ). This pathway requires 2–20 bp of microhomology at or near the break ends, which are subsequently repaired by the DNA polymerase POLQ, frequently resulting in large deletions at the cut site.^10^^,^^11^^,^^12^^,^^13^ In contrast, the HDR pathway leverages an exogenous template to accurately repair the DSB and mediates precise gene correction or the insertion of large transgenes (Figure 2).^6^^,^^14^^,^^15^^,^^16^ Both HDR-based and NHEJ-based genome-editing strategies have been utilized in many pre-clinical and clinical trials for the treatment of hereditary diseases^17^^,^^18^^,^^19^^,^^20^^,^^21^^,^^22^ and malignancies.^23^^,^^24^^,^^25^^,^^26^^,^^27^

The recent development of base editing (BE)^28^ and prime editing (PE)^29^ have expanded the CRISPR toolkit, enabling precise search and replace genome editing without the need for DSB induction. Base editors utilize Cas9 nickase (nCas9), which introduces a single-strand break, fused to a DNA deaminase domain. There are two main classes of BEs, distinguished by the type of deaminase used: adenine base editors, which facilitate the conversion of adenosine to guanine, and cytosine base editors (CBEs), which mediate cytidine to uracil conversion. CBEs typically also include a uracil DNA glycosylase inhibitor domain, which prevents base excision repair and enhances editing efficiency by stabilizing the uracil intermediate. BE allows for the precise correction of transition mutations through single base pair substitutions.^30^ However, BE can still result in unintended bystander deaminations when multiple editable bases fall within the deaminase activity window, potentially leading to additional single-nucleotide changes near the target site. In contrast, prime editors consist of a reverse transcriptase (RT) fused to nCas9, along with a prime-editing gRNA that directs the target site and encodes the intended genetic alteration. Unlike BEs, PEs do not rely on deaminase activity and therefore avoid bystander editing, while also supporting all 12 possible base-to-base swappings, as well as indels, offering greater flexibility compared to BE.^31^^,^^32^

A recent advancement in site-specific knockin strategies involves the use of integrases, particularly serine integrases. These enzymes mediate targeted insertion through a recombinase that contains *attP* attachment site and facilitates recombination at a ∼50-bp *attB* attachment site within the target genome. The programmable addition via site-specific targeting elements (PASTE) method further enhances this approach by incorporating PE to pre-generated *att* sites in the genome, facilitating the integration of large payloads (>100 kb) through integrase-mediated recombination. While more flexible and not restricted to a specific cell-cycle stage, integrase-based methods, unlike seamless HDR, leave residual “scars” in the genome after insertion.^33^

### CRISPR-Cas off-target activity

**Figure 3:**
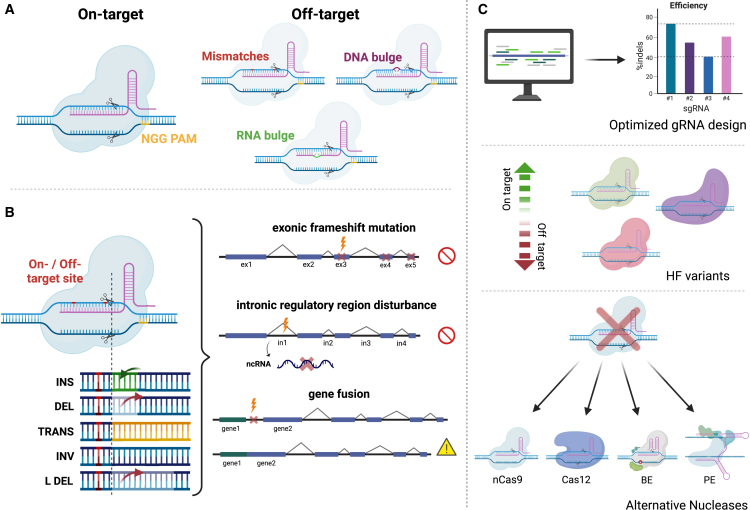
Mechanisms and consequences of OTA (A) Schematic illustration of gRNA/DNA binding at an on-target site (left) and OT sites, which may involve mismatches, DNA bulges, or RNA bulges. Illustration is based on SpCas9. (B) DNA repair outcomes at on-target or OT sites and their potential consequences. Shown are insertion (INS), deletion (DEL), translocation (TRANS), inversion (INV), and large deletion (L DEL). Resulting effects include exonic frameshift mutations, disruption of intronic regulatory regions (e.g., non-coding RNAs [ncRNAs]), and gene fusions. Red lightning bolts indicate potential disruption of gene expression; the yellow caution sign denotes oncogenic potential. (C) Illustration of various strategies to mitigate OT effects, including optimized gRNA design, high-fidelity (HF) Cas variants, and alternative nucleases (nCas9, Cas12, BE, and PE). Created with BioRender.com.

While the CRISPR-Cas system is highly efficient and versatile, adverse editing events at unintended sites, known as off-target activity (OTA), remains a significant safety concern. OTA can occur when the CRISPR-Cas complex associates genomic loci with high sequence identity to the on-target sites, and is further complicated by the tolerance of DNA and RNA base bulges by CRISPR-Cas complexes (Figure 3A).^34^ CRISPR-Cas induced indels at OT sites can disrupt gene regulation and expression, potentially leading to various undesired effects, including oncogenic transformations.^35^^,^^36^ Additionally, substantial genotoxicity arises from larger structural variations (SVs) at both on-target and OTs, including translocations and large deletions, duplications, inversions, partial or full integration of HDR donors, and chromosomal loss (Figure 3B).^37^^,^^38^^,^^39^^,^^40^^,^^41^^,^^42^

In addition to OT mutations, the use of viral vectors for *in vivo* delivery of genome editing components or as HDR donor templates has been associated with unintended genomic integrations of vector or vector-derived fragments. For example, adeno-associated virus (AAV) HDR donors frequently integrate their inverted terminal repeats at both on-target and OT genomic sites.^21^^,^^43^ Furthermore, full-length or fragmented viral vector sequences have been observed to integrate at target genomic loci following *in vivo* delivery.^39^ In primary cells treated with viral HDR donors, concatemeric integrations of the full-length vector have specifically been reported at on-target sites.^44^ Integrations from non-viral double-stranded and single-stranded HDR DNA donors have also been documented, although typically at lower frequencies.^45^

The OTA of CRISPR-Cas systems are influenced by various factors, including the target site sequence, the delivery modality, unequal delivery of the Cas nuclease across treated cells, and the cell type (Figure 3C). A primary determinant is the gRNA sequence itself: gRNAs with unique target sequences, that is with minimal sequence identity to genomic regions other than the on-target, are expected to have a reduced number of potential OTs. Moreover, in most CRISPR-Cas systems, efficient DNA cleavage requires the presence of a protospacer adjacent motif (PAM), specifically 5′-NGG-3′, in the case of the prototypic *Streptococcus pyogenes* Cas9 (SpCas9). In nature, PAMs prevent self-targeting by ensuring that only foreign DNA is recognized and cleaved. In genome editing, this constraint limits the number of accessible genomic sites, but also inherently enhances specificity by reducing the likelihood of OTA. Engineered PAM-relaxed or PAM-less variants expand the range of targetable sites, although this increased flexibility may come at the cost of elevated OTA compared to the PAM-restricted wild-type enzyme.^46^^,^^47^ Chemical modifications on the gRNAs have also reduced OTA by altering the thermodynamic and kinetic properties of the gRNA-DNA heteroduplex formation and promoting increased dissociation rates at OT loci.^48^^,^^49^^,^^50^ Aside from the gRNA spacer itself, the context of the genomic locus, such as epigenetic features and the chromatin state, can influence OTA.^51^^,^^52^^,^^53^ Another key factor is the type of Cas protein utilized, with both natural and engineered Cas variants continuously being discovered and refined to offer enhanced specificity and efficiency.^54^^,^^55^ Recently, large language models have entered protein engineering, including CRISPR proteins.^56^ Optimized editing protocols including the delivery platform (see Cavazza et al.^57^ for a comprehensive review) and gRNA/Cas formulations have also been shown to affect OTA.^58^^,^^59^ Finally, BE and PE aim to minimize OT effects by circumventing the introduction of DSBs. For a comprehensive discussion of strategies to mitigate OTA, we refer readers to the paper by Wienert and Cromer.^35^ Despite these advancements, no current methods have fully eliminated OTA. Even at low frequencies, adverse mutations pose significant risks in therapeutic genome editing, where large numbers of cells are modified, and a single mutated cell could pose a substantial risk. The risk possesed by OTA depends largely on its genomic context. For example, OTAs in proto-oncogenes or coding regions are generally more concerning than OTs within intergenic regions or non-coding regions; however, these can promote the generation of SVs and cannot be overlooked. Therefore, a comprehensive OT analysis remains critical when developing CRISPR-based therapies for clinical use.

**Figure 4:**
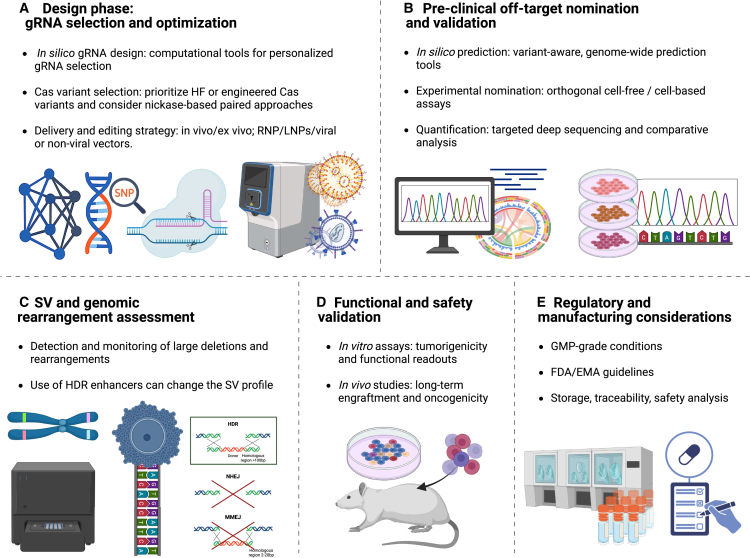
Recommended workflow for CRISPR-based genome editing and genomic safety characterization (A) Design phase: personalized gRNA design using *in silico* prediction tools (e.g., CRISPRme, Cas-OFFinder, COSMID, CRISPRon/off), selection of high-fidelity or engineered Cas variants, and optimization of delivery strategy (e.g., RNP, lipid nanoparticle, viral or non-viral vectors) for *in vivo* or *ex vivo* editing. (B) Preclinical OT nomination and validation: potential OTs are predicted (CRISPRme, Cas-OFFinder, CRISPRoff) and experimentally validated via orthogonal assays (e.g., CIRCLE-seq, Digenome-seq, GUIDE-seq, DISCOVER-seq). Quantification is performed by targeted deep sequencing (e.g., rhAmpSeq) and analyzed with tools like CRISPECTOR or CRISPResso2. (C) SVs and genomic rearrangement assessment: monitoring of large deletions, translocations, and other rearrangements using designated methods such as CAST-seq, PEM-seq, ddPCR, FISH, long-read sequencing. (D) Functional and safety validation: edited cells are evaluated via *in vitro* assays (cytotoxicity, tumorigenicity, differentiation, lineage tracking) and/or *in vivo* xenotransplantation into humanized mouse models to evaluate long-term safety, engraftment, and functional performance. (E) Regulatory and manufacturing considerations: all analyses are conducted under GMP conditions, using orthogonal methodologies aligned with FDA/EMA guidelines, with traceable data archiving for potential retrospective safety review. RNP, ribonucleoprotein; EMA, European Medicines Agency; FDA, US Food and Drug Administration. Created with BioRender.com.

OTAs should be monitored carefully throughout the various stages of gRNA design, pre-clinical studies, and clinical studies (Figure 4). As a first step, gRNAs with minimal predicted OTA should be selected while considering genetic variations across populations. Once a gRNA or combination of gRNAs is chosen, potential OTs should be identified and assessed for actual damage under clinically relevant conditions. If adverse OT mutations are discovered, their functional consequences must be assessed thoroughly, with a particular focus on their potential to induce tumorigenicity. This review provides an overview of the tests and assays essential for evaluating the safety of genome-editing therapies for clinical applications and outlines the challenges and benefits of available tools and methods for each stage of the process, focusing on CRISPR-Cas9 applications. Of note, OTA can also be triggered by factors other than the gRNA. For instance, in the case of BE, nonspecific deamination of DNA or RNA can lead to unintended modifications. While this review focuses primarily on CRISPR-Cas9-based nucleases, we recommend further reading on other classes of genome editors.^60^^,^^61^

## *In silico* methods for gRNA design and selection

### The concept of gRNA design

The first step in genome-editing experiments is to design an efficient and specific gRNA with maximal on-target efficiency and minimal OT potential. To do this, *in silico* methods are used to identify typically multiple or several on-target gRNA candidates that meet these on-target and OT criteria. When conducting knockout (KO) experiments of protein-coding genes, the strategy is to target the first couple of exons causing random edits leading to nonsense-mediated decay or dysfunctional proteins serving as KO. For non-coding RNAs, the strategy is typically to employ two gRNAs and remove the entire gene.^62^ Whereas KO studies allow for a pool of gRNAs to select from, specific edits such as a single genomic location often have limited gRNA possibilities—for example, by requiring the presence of a PAM or, in the case of BE, the availability of adenosine or cytosine bases. Hence, “gRNA design” is a matter of selecting gRNAs based on how well they meet the criteria. To meet the design criteria, computational prediction methods are applied. Computational methods for gRNA design generally fall into two main categories. The first category is sequence-only methods, which scan reference genomes to identify candidate gRNAs that meet specified requirements, such as target region or PAM sequence, and detect potential OTs with sequence similarity to the intended target. For the OT location detection, these methods employ sequence similarity to the gRNA. The second category is methods that score a potential on-target or OT site. This can be done by (1) hypothesis-driven algorithms, which apply established biological principles (e.g., mismatch position or kinetic modeling) to predict gRNA performance (see, for example, Eslami-Mossallam et al.^63^), and/or (2) machine learning (ML)-based algorithms, trained and tested on experimental datasets to predict cleavage efficiency at on-target and OT sites. ML methods are inherently limited by the quality and scope of their training data. The choice of computational method and training procedure may provide further limitations beyond those already present in the training data.

### Datasets for building machine learning models

Various types of experimental datasets are available in the public domain, predominantly concerning SpCas9 cleavage results. These contain hundreds to tens of thousands of gRNAs for which their on-target efficiencies and/or corresponding OTAs have been measured by different experimental strategies either *in vitro* or *in vivo*. Early studies focused on generating loss-of-function data to train models using a functional readout from KO of coding genes (e.g., Doench et al.^64^). Most current datasets are indel based using high-throughput sequencing to measure the actual editing activity.^65^^,^^66^^,^^67^^,^^68^ The resulting reads are mapped to their respective genomic locations to call on-target and OT events, using various versions of mapping and sequence alignment algorithms. Hence, the mapping and sequence alignment tools must be carefully evaluated prior to selecting the data to build prediction models, as any errors introduced at the data-generation step would directly affect the prediction outcome.Additionally, ML methods trained on one dataset and evaluated on an external dataset are not expected to perform better than the correlation between them. Some of the datasets show moderate correlation (Spearman R ∼0.5–0.7),^69^ but not strong enough to ensure consistent performance across studies. Therefore, expectations for model performance on external datasets should be adjusted accordingly. Of note, each experimental dataset is generated in a specific cell type and may not be accurately transferred to others, as different cell types, editing modalities, and the active fractions of gene editing nucleases present in a given biological system can contribute to distinct editing profiles.^65^ Another challenge is that large-scale datasets, for example by Lazzarotto et al.,^65^ are far from covering the full mismatches space. Questions remain whether the observed gaps are due to limitations in data accuracy or underlying biological factors.

### Prediction of on-target and OT activities

Several *in silico* methods have been made for both on-target and OT prediction using the above-mentioned datasets for training. The core concept is to assign scores to given on-target or OT sites that correlate with experimental activity data, such as log-transformed read counts from indel-based assays. Many different methods exist for predicting gRNA activity and the repair outcome of different Cas enzymes in different organisms, and while it is beyond the scope of this paper to provide a full overview, we emphasize some of the key methods below representing the main steps in the development of computational strategies.

For on-target efficiency prediction, a few nucleotides of its up- and downstream contexts are used as input to a computational method. For indel-based datasets, efficiency is measured as the fraction of reads carrying an edit. Input data can be featurized (for example, by counting specific dinucleotides and noting nucleotide positions), or it can be one-hot or sparsely/one-hot encoded, where each nucleotide in the target sequence is represented by a binary vector (one “1” and three “0s”). Whereas the former often is used for more “classical” ML and tree-based methods such as gradient boosting tree regression, the latter is most often used for machine/deep learning methods.

While the earlier methods for on-target efficiency prediction were based on functional readouts with the Azimuth package,^64^ a major advance for Cas9 gRNA on-target efficiency prediction was the DeepHF and DeepSpCas9 methods,^70^^,^^71^ which introduced large-scale, indel-based data followed by deep learning strategies for gRNA efficiency prediction. Whereas DeepHF's training data was biased toward highly efficient gRNAs, the DeepSpCas9 used a more balanced gRNA efficiency distribution, making it more compatible with new datasets. Xiang et al. subsequently integrated these data into CRISPRon, establishing a state-of-the-art model for Cas9 on-target efficiency prediction, validated on external, independent test sets.^69^ Notably, although these methods are based on deep learning, more classical ML methods, such as gradient-boosting regression trees, perform relatively close to the deep-learning based methods with the currently available data.^69^

Similar to on-target efficiency prediction, OT cleavage efficiency (activity) prediction is also a matter of scoring. However, before scoring potential OT regions, they need to be located. The simplest approach assumes no bulges in gRNA-DNA interactions, identifying OTs based solely on mismatches and enabling fast search with basic alignment algorithms. However, single-base “bulges” are often tolerated by Cas9 and allow OTA.^34^ Some tools that search for putative OTs do allow for bulges. The COSMID tool, for example, permits the identification of sites containing one base indel within the target sequence using a so-called tree-like data structure.^72^ Cas-OFFinder uses a more exhaustive search anchored in the PAMs and allows up to two bulges in gRNA or DNA.^73^

Some of the prediction tools do not provide a scoring, but merely locate potential OTs and the possible configurations of the mismatches and bulges within the spacer:DNA interaction. Scoring OTs solely on the number and position of mismatches and bulges is not advisable, as it has been demonstrated that single mismatches may reduce activity far more than multiple mismatches in some contexts.^66^ Furthermore, the distance of a gRNA:DNA mismatch from the PAM sequence impacts the level of OTA, with mismatches closer to the PAM generally having a more disruptive effect than distal mismatches.^36^^,^^74^^,^^75^ Mismatches within the PAM are also tolerated to some degree, with SpCas9 capable of recognizing the alternative PAM NAG.^75^ This has also been observed with other orthologs such as the *Streptococcus thermophilus* Cas9, which has a canonical PAM of NNAGAAT but can tolerate mismatches at positions 3, 4, 5, and 7.^76^ Therefore, alternative PAM sequences need to be considered when developing *in silico* prediction tools or when mapping reads from cell-based assays. Moreover, a recent *in silico* analysis supported by experimental results showed that, for SpCas9, the nucleotide context around the PAM strongly impacts gRNA efficiency, with Gs upstream of the PAM enhancing efficiency, whereas Gs downstream of the PAM tend to reduce activity.^77^ This positional effect partially explains why sequence context contributes to gRNA activity prediction. Furthermore, including the gRNA:DNA binding energy in the deep learning scheme for gRNA efficiency prediction was shown to improve the prediction accuracy,^69^ consistent with the observation of a sweet-spot binding energy interval in which the gRNAs have the highest efficiency.^77^ Additional parameters that have also been incorporated into scoring metrics include the nature of the mismatch,^75^ Cas9 binding data from chromatin immunoprecipitation,^53^^,^^78^ and DNA accessibility.^79^

Based on approaches that search for potential OT locations, a number of mainly ML algorithms have been developed.^64^^,^^75^^,^^79^^,^^80^^,^^81^^,^^82^^,^^83^^,^^84^^,^^85^^,^^86^ ML-based prediction tools generally outperform simpler baseline models in OT prediction. However, a binding-energy-based model, CRISPRoff,^87^ which approximates biophysical interactions within the Cas9-gRNA-DNA complex, surpassed top machine learning models of the time and continues to outperform them in some comparisons, as shown in recent benchmarks by Chen et al.^88^ Baseline models like CRISPRoff are simple and incorporate basic, interpretable properties, in contrast to ML methods, which despite advanced feature extraction, lack straightforward interpretability. Furthermore, mechanistic models like CRISPRoff are less dependent on organism-specific data, unlike ML models, which often rely on such data for training. The observation that simpler models, such as CRISPRoff, can sometimes outperform more sophisticated machine learning approaches is intriguing. We propose two explanations: (1) current training datasets may lack sufficient diversity for complex models to learn beyond what simpler rule-based algorithms already capture, and (2) the biological mechanisms driving OTA may be relatively simple and already well approximated by energy-based approaches. Without broader empirical data, it is difficult to determine which explanation is more accurate.

The recent CRISOT framework takes features of molecular dynamics into account in combination with a machine-learning framework.^88^ Whereas these methods mainly focus on mismatch patterns, only CRISPR-Net^89^ scores with bulges, using the locations and gRNA spacer:DNA interaction configurations.

Finally, many of the tools described above are available through web interfaces, including COSMID, CRISPOR, CHOP-CHOP, CRISPRon/off, PE-Designer, and PnB-Designer.^72^^,^^90^^,^^91^^,^^92^^,^^93^^,^^94^^,^^95^^,^^96^

### Benchmarking *in silico* OT identification tools

Some review papers have attempted to provide a comprehensive overview of computational OT prediction, including reporting on the performance of a subset of available methods.^97^ However, these reviews face challenges in their benchmarks by blindly referencing performance reports from individual papers without ensuring that the methods are compared on identical datasets. Therefore, such comparisons are not informative in terms of deciding which methods to use. The overview of the different methods can, however, help users to obtain an overview of the different approaches.

When developing methods, especially ML-based methods, full evaluation of independent data is critical. Generally, a method's performance on external datasets will be limited by how closely the external data correlate with the training data (see above). This is also why benchmarking on external datasets often yields lower performance than evaluation on held-out data from the same experiment. Hence, a full evaluation of independent data is critical.^98^ For training, validation, and testing, this is more straightforward as all the OTs accompanying a gRNA can be used as independent held-out sets (assuming the gRNAs differ substantially). However, gRNAs vary widely in the number of associated OTs, and from a therapeutic perspective, there may be greater interest in algorithms that perform well on gRNAs with fewer OTs.

In addition to using publicly available datasets as external, independent OT references, Bao et al. generated a new dataset of validated OT sites from scratch.^99^ Evaluation of several computational methods revealed lower performance when tested on the novel set of validated OT sites from four gRNAs, compared to a dataset of published OT sites from 27 gRNAs that were part of the training data, demonstrating that reported model performance is likely overstated. Larger, unique datasets are required to confirm this observation, while other factors, such as batch effects of data generated in different labs, cannot be ruled out.^99^

In summary, computational pipelines for selecting optimal gRNAs have advanced significantly and continue to evolve with the integration of new data and models, serving as a valuable starting point for identifying candidate gRNAs. However, as evident from the evaluations of the computational methods mentioned above, *in silico* tools still exhibit high false positive rates and can predict thousands of OTs per gRNA, while the data typically suggest much fewer ones. This challenge is in part attributed to the limitations of the available data and in part to the OT location identification and scoring. Current experimental data used in these models do not fully account for three-dimensional genome structure or the molecular context near potential OTs, nor do they do so for on-target efficiency data. Furthermore, current computational tools for gRNA design are largely limited to well-characterized nucleases like SpCas9 and their standard PAMs, with limited applicability to novel or engineered CRISPR systems. These models typically assume a fixed gRNA scaffold and are trained on data from a narrow range of cell types (typically HEK293T or similar), reducing their generalizability. As a result, computational predictions should be considered a useful first filter, but empirical validation remains essential for selecting the most effective and specific gRNAs. Thus, a dataset with a higher number of potential false positives for subsequent experimental testing is preferable to one that might prematurely exclude true OTs. For the field to advance further with more accurate computational methods, we need even better data than those available today taking effectively as much of the cell state into account as possible.

## Experimental assays for OT sites nomination

### Cell-free and cell-based on-target nomination assays

Experimental assays for OT identification can be broadly classified as cell-free (*in vitro*) or cell-based assays (see Tables 1 and 2). Early cell-free methods made use of synthetic DNA libraries containing up to 10^12^ sequences covering all permutations of a target sequence with ≤8 mismatches.^113^ This was followed by several studies that used extracted genomic DNA as a substrate for Cas9 cleavage^65^^,^^67^^,^^100^^,^^102^^,^^103^ with different approaches to enrich for Cas9 cleaved products from a background of randomly sheared DNA, including biotinylated adaptor ligation,^102^ and circularization of DNA fragments.^67^ These cell-free studies allowed for a great degree of control of the reaction parameters as it is relatively straightforward to modify the enzyme (Cas9) and substrate (DNA) concentrations as well as the reaction time. Each of these parameters greatly influences how many potential OTs are identified, with increased time and Cas9 concentration resulting in more potential OTs being identified, although these additional sites generally have lower signals and fail to validate in follow-up targeted amplicon sequencing studies. The failure of these sites to validate could indicate that they are false positives or that they occur at a frequency below the limit of detection of high-throughput sequencing (∼0.1%).^67^ The *in vitro* assays are agnostic to cell-based factors or chromatin structure, which potentially allows for a broader scope of OT identification and more data for developing OT prediction models. The use of synthetic DNA libraries can also be used to identify variant-sensitive OTs facilitating the assessment of OTA in the context of global human genetic variation,^104^ but for clinical translation it may be more beneficial to assay therapeutic gRNAs in a relevant cell model with appropriate concentrations of the gene-editing components. For schematic overviews of key detection platforms and workflows, we refer readers to a review by Cavazza et al.,^114^ which provides clear illustrations of both *in vitro* and cell-based OTA detection strategies.

**Table 1:** *In vitro* OT nomination assays

Method	Description	Advantages	Disadvantages
Digenome-seq^100^	*in vitro* Cas9 digestion, followed by next-generation sequencing (NGS) and computational analysis to detect indels	genome-wide; sensitive	requires high sequencing depth; high false positive rate; lacks the chromatin context
DIG-seq^101^	modified version of Digenome-seq using chromatin DNA	genome-wide; sensitive; chromatin context	requires high sequencing depth
SITE-seq^102^	*in vitro* Cas9 digestion; biotinylated adapters are then added to the cleaved ends; biotin selection and PCR amplify the target region for NGS	genome-wide; sensitive	requires high sequencing coverage; lacks the chromatin context
CIRCLE-seq^67^	*in vitro* Cas9 digestion of circularized genomic DNAonly circular DNA containing nuclease cleavage sites is linearized and utilized for NGS library preparation	genome-wide; sensitive	requires large amounts of DNA; lacks the chromatin context
CHANGE-seq^65^	a modified version of CIRCLE-seq that utilized enzymatic fragmentation instead of sonication	genome-wide; sensitive; lower amount of input DNA	expensive compared to CIRCLE-seq; lacks the chromatin context
RGEN-seq^103^	*in vitro* Cas9 digestion followed by the ligation of the cut ends to a truncated NGS adapter that lacks the flow-cell binding site; a complementary sequencing adapter is then added in a subsequent step, allowing for the sequencing of only the cleaved DNA	genome-wide; sensitive; simplified workflow compared to other cell-free assays; amplification free	requires high sequencing depth; lacks the chromatin context
ONE-seq^104^	*in vitro* Cas9 digestion in synthesized oligonucleotide libraries	enables testing thousands of sites in parallel; supports variant-aware analysis	limited to pre-defined regions; lacks the chromatin context

**Table 2:** *In vivo* OT nomination assays

Method	Description	Advantages	Disadvantages
BLESS^105^	following CRISPR-Cas editing, cells are fixed, and nuclei are purified; DSB are captured using a biotinylated linker, allowing for their enrichment and subsequent sequencing	genome-wide; can be applied to *in vivo*-derived tissues	low sensitivity and high background; requires cell fixation; requires high cell input; centrifugation steps in the protocol shear the DNA and introduce background
BLISS^106^	following CRISPR-Cas editing, cells are fixed onto a microscope slide; DSBs are ligated to an adapter containing T7 promoter and sequencing adapters; DNA is amplified using *in vitro* transcription, followed by NGS	genome-wide; higher sensitivity compared to BLESS	requires cell fixation; low sensitivity
IDLV integration^107^	cells are edited in the presence of Integrative-Deficient Lentiviral Vectors (IDLV) vectors, which integrate the DSBs; linear-amplification mediated (LAM)-PCR is then performed using primers complementary to the IDLV sequences, followed by NGS	genome-wide; applicable to various cell types/nucleases	requires viral vector transduction; low integration bias
AAV integration^39^	cells are edited in the presence of AAV vectors, which integrate into the DSBs; PCR amplification is then performed using primers complementary to the AAV sequences, followed by NGS	genome-wide; applicable to various cell types/nucleases	requires viral vector transduction; low integration bias
GUIDE-seq^66^	cells are edited in the presence of a double-stranded oligonucleotide (dsODN), which integrates into the DNA breaks; PCR amplification is then performed using primers complementary to the DNA tag, followed by NGS	genome-wide; high sensitivity	some cells are sensitive to the dsODN (e.g., HSPCs); compatible only with nucleases that generate blunt ends at the DSBs, such as SpCas9
OliTag-seq^108^	optimized version of GUIDE-seq with a more stable tag and enhanced PCR amplification	genome-wide; high sensitivity	some cells are sensitive to the dsODN (e.g., HSPCs)
DISCOVER-seq^109^^,^^110^	chromatin immunoprecipitation (ChIP-seq) targeting MRE11, a key protein involved in the DSB repair mechanism, followed by NGS	can be also applied *in vivo*; high specificity	lower resolution compared to other cell-based methods; requires cell optimization
DISCOVER-Seq+^109^	optimized version of DISCOVER-seq using DNA-protein kinase catalytic inhibitor to achieve higher MRE11 levels	higher sensitivity compared to DISCOVER-seq	requires NHEJ inhibition, also in *in vivo* settings
INDUCE-seq^111^	PCR-free, ligation-based method that labels DSBs in fixed nuclei, followed by direct sequencing to digitally quantify break sites	genome-wide; reduces amplification bias; high sensitivity and resolution	requires cell fixation; limited compatibility with certain cleavage profiles
SURRO-seq^68^	transduction of synthesized lentiviral libraries containing custom OT sites and site-specific barcodes, followed by NGS	enables testing thousands of sites in parallel; supports variant-aware analysis	limited to predefined regions; requires viral vector transduction; background noise due to errors in the library synthesis
ABSOLVE-seq^112^	transduction of synthesized lentiviral libraries containing custom OT sites and site-specific barcodes with unique molecular identifiers (UMI), followed by NGS	enables testing thousands of sites in parallel; supports variant-aware analysis; computational pipeline for noise filtering; contains UMI for improved quantification	limited to predefined regions; requires viral vector transduction; background noise due to errors in the library synthesis; complex computational pipeline required to handle the data

Cell-based assays detect OTA in CRISPR-Cas-treated cells via the identification of DNA breaks,^106^^,^^109^^,^^110^^,^^111^ repair products such as indels or translocations,^40^^,^^68^^,^^115^^,^^116^ or the integration of exogenous DNA, such as virus vectors or short DNA tags, into the DNA break.^39^^,^^66^^,^^107^^,^^108^ The use of integrase deficient lentiviral vector capture into DNA breaks has been used to map nuclease OT sites in human cells but suffers from low sensitivity and can typically detect frequently cut OT sites with >1% indel formation.^107^^,^^117^ When short double-stranded DNA tags are used for DSB capture, the sensitivity of the approach is limited by the error rate of current sequencing technology (0.1%).^66^

Unlike methods that detect repair outcomes (indels or DNA integration), those that detect DNA breaks in cells are time sensitive as they cannot detect OTA once the DNA breaks have been repaired. This can lower their overall sensitivity; however, time-resolution studies using these approaches could provide invaluable data on Cas9 kinetics within human cells. The sensitivity of these methods that are reliant on detecting DNA breaks can be dramatically improved by inhibiting NHEJ DNA repair, which increases the availability of DNA DSB ends for detection.^109^

Other cell-based methods that rely on the detection of structural variants such as translocations induced by OTA are discussed further in the section “detection of adverse genomic rearrangements.”

In addition to the *ex vivo* experimental assays, animal models are sometimes used to evaluate editing specificity *in vivo*, particularly when assessing long-term effects or biodistribution.^118^ While these systems can provide important safety insights, it is important to acknowledge their limitations, as many human-specific DNA sequences are absent in these models, which may restrict the detection of clinically relevant OT effects. For a more comprehensive discussion of these considerations and the limitations of animal models in genome editing research, we refer readers to Soufizadeh et al.^118^ and Gopinath et al.^119^

### Benchmarking experimental OT nomination assays

In studies that report cellular validation via deep sequencing of potential OTs, the false positive rates varied from 20% to 95%,^99^ indicating that either these experimental assays suffer from high false positive rates or that current sequencing technology is not sensitive enough to detect OTs with low editing rates.^67^ Improved sensitivity of deep sequencing for indel detection has been described using oversampling of input genomes, which in extreme cases comprise a single amplicon for an entire NextSeq run and improved sensitivity 100-fold.^120^

Many of these experimental tools have been developed to detect rare OT events and provide a worst-case scenario using DNA overexpression systems.^59^ How this compares to OT effects in primary cells of interest under Good Manufacturing Practice (GMP) conditions with CRISPR ribonucleoprotein complexes would also be useful for supporting the translation of CRISPR-based medicines to the clinic, and the differences in OT effects seen with different expression systems (DNA, mRNA, protein) could provide valuable information on the kinetics of OT editing.^109^^,^^121^

No formal benchmarking or direct comparison of the various experimental tools available using a set of standard gRNA sequences has been carried out, making it difficult for researchers to choose the most appropriate assay. These assays require specialized expertise in addition to significant investment and resources to establish in the lab, and a direct comparison using a set of validated gRNAs with known OT sites would not only allow for a fair and unbiased quantification of the true and false positive rates of each assay but also help researchers make an informed decision on which assays to use. Validation of OTA across different tools would also facilitate the creation of a database of validated OTs that could serve as a ground truth dataset to aid in the development of improved machine learning tools for OT identification. In the absence of a single gold standard assay, applying a combination of orthogonal approaches remains the most effective strategy.

### Quantifying OTA

Since no single method can fully capture the OT landscape, regulatory bodies require the use of multiple orthogonal techniques from the wide array of *in silico*, cell-free, and cell-based approaches available (https://www.fda.gov/media/156894/download). Once a final list of potential OT sites is compiled, the presence of indels is quantified to address the level of activity of each nominated site. A state-of-the-art approach utilizes targeted amplification, such as the rhAmpSeq method,^122^ followed by deep sequencing, enabling the detection of even rare editing events. Ideally, this should be performed under conditions that simulate the clinical manufacturing process. The data are then analyzed in one of many available tools.^42^^,^^123^^,^^124^^,^^125^^,^^126^ CRISPResso2^123^ is widely used and offers various options, including specific analysis for different types of Cas enzymes and analysis of HDR donor integration rates. However, CRISPResso2 does not support direct comparison with untreated samples, resulting in high background control. CRISPECTOR^42^ uses a Bayesian classifier to analyze treated CRISPR-Cas9 samples against an untreated control sample, providing an accurate and statistical approach to assess editing levels. Furthermore, CRISPECTOR can detect translocations between the on-target and OT sites amplified.

Despite its sensitivity, the targeted PCR approach has several limitations, including susceptibility to PCR-induced bias, amplification challenges in certain genomic regions, and technical difficulties in scaling to panels exceeding 200 sites. An alternative solution is using hybridization-based technologies, such as Capture-seq,^127^ which leverages custom-designed probes to selectively enrich target regions of interest. Unlike PCR-based amplification methods, Capture-seq does not rely on primer binding or amplification efficiency, thereby reducing amplification bias and enabling more uniform coverage across OTs. This PCR-free approach is particularly valuable for accurate quantification of low-frequency editing events and has been applied in clinical studies such as Vertex’s exa-cel trial.^128^ Another approach for quantifying OT effects is utilizing RNA sequencing (RNA-seq) data. CRISPRroots^129^ integrates DNA:RNA binding properties, gene expression changes, and sequence variants to identify and prioritize potential OTs in Cas9 edited samples. However, its applicability is restricted to coding regions.

## Personalized, variant-specific OT studies

### The need for personalized genome editing

The human genome is diverse across populations and individuals, due to millions of single-nucleotide polymorphisms (SNPs), indels, and copy-number variants.^130^^,^^131^^,^^132^^,^^133^ Nevertheless, most computational pipelines for gRNA design, OT prediction, and OT analysis rely on a single consensus reference genome, typically GRCh37 (hg19) or GRCh38 (hg38). Genomic variants in CRISPR-Cas on-target or OT sequence can alter the binding efficiency of the nuclease and hence affect the efficiency or specificity of editing.^134^^,^^135^ Moreover, many of the CRISPR-Cas therapies are directed at diseases that are genetically enriched within specific populations, further underscoring the importance of considering genomic variations in therapeutic design. A recent study by Cancellieri et al. demonstrated that a Cas9/gRNA targeting the *BCL11A* enhancer used in Casgevy, the first approved CRISPR-based therapy, showed activity at an OT site specific to individuals of African ancestry as a result of a SNP creating a novel PAM site.^136^ In addition to affecting gRNA binding and OT profiles, SNPs can also impact the on-target integration efficiency of HDR-based strategies, as sequence mismatches within the homology arms have been shown to affect HDR efficacy.^137^ While viral HDR donors with ∼200- to 1,000-bp arms and single-stranded DNA donors with ∼30- to 100-bp arms generally perform well across individuals, in cases where high-frequency SNPs or deletions overlap the homology arms, personalized donor templates are required to ensure efficient and accurate editing.

The need for variant-specific OT assessment was acknowledged by regulators, who require researchers to address the potential impact of genetic heterogeneity on drug safety (https://www.fda.gov/media/171593/download).

### Variant-aware OT prediction and measurement

In recent years, several large-scale projects have generated extensive sequencing data to provide a more accurate representation of human genome diversity. Databases such as the 1000 Genomes Project,^138^ gnomAD,^139^ and dbSNP^140^ offer comprehensive estimates of SNP distribution across diverse populations. Moreover, the recently developed pangenome reference sequence, which integrates assemblies from a genetically diverse cohort, enhances variant-aware OT site prediction.^132^ CRISPRme^136^ utilizes public databases for variant-aware OT prediction, accepting variant data from various databases and population-specific or individual VCF files. Individual information is particularly important when working with underrepresented populations in genomic studies or when developing therapies for founder mutations.

Variant-specific predictions often yield a large number of potential OTs, posing a challenge for confirmation through targeted sequencing approaches. A promising approach to address this challenge is the use of synthetic libraries that encompass all relevant variants, enabling high-throughput screening of multiple sites simultaneously. The *in vitro* ONE-seq^104^ assay, for example, leverages synthetic DNA fragments to estimate the CRISPR-Cas cleavage frequencies in different predicted OTs. While offering simplicity, *in vitro* assays lack the cellular context. In contrast, SURRO-seq^68^ and ABSOLVE-seq^112^ assays involve transducing cells with lentiviral libraries encoding various sites and measuring CRISPR-Cas cleavage frequencies in live cells. These methods have shown a strong correlation with editing rates in endogenous loci but suffer from a high signal-to-noise ratio and may be inaccurate, particularly for OT effects where editing occurs at levels below 1%.^112^

Following OT nomination, targeted deep sequencing in primary cells relevant to the specific therapeutic application remains the most accurate method for OT assessment, particularly when conducted under GMP-grade conditions. To account for genetic variation, sequencing data should be analyzed in the context of each sample’s unique genome sequence. CRISPResso2 supports allele-specific OT analysis for CRISPR-Cas experiments, including BE, PE, and HDR integration.^123^ However, it requires prior information about the sample’s genotype. The recently developed CRISPECTOR2.0^141^ software offers allele-specific quantification of CRISPR-Cas activity, contains a built-in allele-calling feature, and does not require prior genotype information.

The field of genome therapy is moving rapidly toward a more personalized approach, enhancing the safety and precision of treatments. The question remains whether allele-specific analysis should be population specific or individual specific. Population-specific approaches, which account for common variants within defined groups, offer a practical and cost-effective solution. They can be broadly applied, reduce resource demands, and improve accuracy over reference-only methods, although they may miss rare or private variants. In contrast, patient-specific analysis captures the full spectrum of individual variation, offering the highest precision, but at the cost of scalability, as it requires collecting a specimen from the specific patient or disease case to enable variant-aware OT prediction and editing strategy design. Such personalized analysis also poses regulatory challenges related to standardization and raises concerns about equitable access, especially in resource-limited settings. Ultimately, the choice between these approaches will depend on balancing precision with practical considerations of cost and feasibility.

## Detection of adverse genomic rearrangements

### CRISPR-Cas induces SVs

It is widely known that designer nucleases relying on DSBs often result in localized genomic sequence alterations, mainly indels derived from the NHEJ repair pathway. However, no matter how precise, a DSB is still a lesion that can trigger other DNA damage responses (DDRs), potentially resulting in more complex genomic resolutions, such as SVs.^142^^,^^143^^,^^144^^,^^145^^,^^146^^,^^147^^,^^148^^,^^149^^,^^150^ These aberrations consist of inter- and intra-chromosomal rearrangements, including inversions, large indels, and duplications.^40^^,^^149^^,^^151^^,^^152^ The biological consequences of these outcomes vary depending on their frequency and the extent of the affected region. For instance, large deletions can not only span several megabases but also result in chromosomal truncation or complete loss of a chromosome.^38^^,^^40^^,^^142^^,^^143^^,^^152^^,^^153^ Any large deletion can lead to complete gene loss if both homologous chromosomes are affected or to hemizygosity if only one is involved. The latter case presents various functional repercussions depending on gene dosage requirements, such as a neutral phenotypic effect, haploinsufficiency, loss of heterozygosity, or loss of imprinting.^142^

From a molecular standpoint, collateral editing at OTs is not different from that at the on-target site and can therefore induce the same type of SVs. However, simultaneous DSBs at different loci add the possibility of reciprocal translocations, not only between target and OTs but also between different OTs. In some cases, these rearrangements can generate other types of unbalanced translocations such as dicentric or acentric chromosomes (with the latter product generally being lost during subsequent mitoses) or more convoluted scenarios like chromothripsis.^40^^,^^146^^,^^154^ Unintentional targeting of a proto-oncogene or a tumor-suppressor gene is not the only risk potentially leading to carcinogenic development of the cell product. Even OT-driven reciprocal balanced translocations, without any loss of genetic material, could ultimately result in tumorigenesis, since, for example, a fusion of a regulatory element to a proto-oncogene has been reported to be a driver of malignant transformation.^155^^,^^156^^,^^157^^,^^158^^,^^159^ Until recently, SVs have often been overlooked, yet it is evident that their detection and characterization are critical for ensuring the safety and efficacy of gene-editing applications.

### Detection of adverse SVs in genome-engineered cells

The identification and characterization of SVs derived from gene editing can be challenging, considering their diversity and sometimes low-frequency occurrence. Several methods allow their detection, which can be broadly categorized into biased and unbiased (or less biased, to be precise) assays (Table 3). Biased assays rely on previously known candidate sites. For instance, long-read sequencing can be used to outline the pattern of large deletions surrounding a region of interest.^146^^,^^149^^,^^152^^,^^164^ This approach can only be conducted within a pre-defined range, and the quantification of deletions should be carefully interpreted due to the preferential amplification of smaller over larger fragments. This problem could be overcome by using amplification-free capture methods, although this would require large amounts of input material. Conventional or digital droplet PCR (ddPCR) allows for straightforward detection of translocations involving the on-target and any suspected/predicted OTs by designing primers flanking the junctions.^37^^,^^38^^,^^165^^,^^166^ However, a concomitant sequence alteration preventing primer hybridization could yield a false negative result. Other techniques like fluorescence *in situ* hybridization (FISH), optical genome mapping (OGM), or comparative genomic hybridization (CGH) arrays offer additional probe-based detection methods for SVs, both in biased and unbiased contexts. The lower limit of detection of these methods, however, would not be enough to confidently discriminate against rare events from background signals.^168^^,^^169^^,^^170^^,^^171^

**Table 3:** SV detection assays

Method	Description	Advantages	Disadvantages
**Long-read sequencing**			

targeted long-read sequencing^151^^,^^152^^,^^160^^,^^161^	targeted PCR amplification followed by long-read sequencing using Oxford Nanopore Technologies (ONT) or Pacific Biosciences (PacBio)	capable of detecting large, complex, and repetitive SVs	limited to detection of known SVs; not suitable for genome-wide detection; PCR amplification bias
SMRT-OTS, NANO-OTS^162^	amplification-free long-read sequencing for PacBio and ONT, respectively	amplification-free	higher error rate compared to short-read sequencing; high cost and longer run times; requires large amounts of DNA
Cas9 enrichment^163^	amplification-free enrichment using CRISPR-Cas9 with gRNAs at both ends of the target region	amplification-free	limited to targeted regions; not suitable for genome-wide SV detection; requires prior knowledge of target loci
LongAmp-seq/long-range amplicon sequencing^149^^,^^164^	optimization of long-range PCR and Illumina-based sequencing to quantify SVs	based on short-read sequencing; cost-effective	limited to predefined regions; amplification bias may miss some SVs

**Probe-based methods**			

ddPCR^37^^,^^38^^,^^165^^,^^166^^,^^167^	uses droplet microfluidics to partition samples into droplets, each containing a single DNA molecule labeled with fluorescent probes	absolute quantification of specific, known SVs	limited to predefined regions; amplification bias; may miss some SVs
FISH, FISH-IS^168^^,^^169^	uses fluorescent probes that bind to specific DNA sequences to visualize SVs, typically in condensed chromosomes	direct visualization of chromosomal rearrangements; captures large genomic rearrangements	low resolution compared to sequencing; labor intensive; requires microscopy
OGM^170^^,^^171^	OGM uses restriction enzymes to label DNA with unique fluorescent barcodes, which are then imaged and analyzed to create physical genome maps	detects a wider array of SVs and CNVs; amplification-free.	limited resolution for small SVs; lower throughput compared to sequencing-based methods; requires specialized equipment
array-CGH^148^	genome-wide DNA copy-number analysis is performed based on the relative intensity of complementary fluorescent probes between sample and control DNA	can detect SVs that result in copy-number gain or loss	limited to detecting CNVs; unable to detect balanced SVs like inversions or translocations; low resolution; low sensitivity

**Bait-prey assays**			

LAM-HTGTS, iHTGTS^115^^,^^172^^,^^173^	LAM-PCR with biotinylated primer, located in proximity to a bait genomic region	genome-wide; inexpensive and scalable	detects only chromosomal rearrangements with the on-target (bait) site; requires large amounts of DNA; requires complex bioinformatics analysis
PEM-seq^174^	primer extension with biotinylated primer, located in proximity to a bait genomic region	genome-wide; eliminates amplification noise	detects only chromosomal rearrangements with the on-target (bait) site; requires complex analysis; may miss small SVs or those in repetitive regions
UDiTaS^116^	tagmentation with a custom Tn5 transposon, followed by an amplification using a bait primer	enzymatic fragmentation instead of sonication	limited to predefined loci; requires prior knowledge of target regions; not suitable for genome-wide analysis
CAST-seq^40^	bait-prey strategy using decoy primers for an increased sensitivity	genome-wide; also detects on-target aberrations; higher sensitivity (0.01%) compared to other bait-prey assays	detects only chromosomal rearrangements with the on-target (bait) site; requires complex bioinformatics analysis

When it comes to previously unidentified sites, HTGTS, UDiTaS, PEM-seq, and CAST-seq are methods capable of genome-wide detection of some species of SVs using an “anchor” or a “bait” genomic region.^40^^,^^115^^,^^152^^,^^172^^,^^175^ All these tools share a common principle based on using a primer or probe located near the DSB at the on-target site as bait to capture rearrangements with another locus. To improve sensitivity, CAST-seq uses decoy primers to inhibit the capture of unedited sequences, thereby increasing the detection limit of aberrant events to as low as 0.01%. In addition, the CAST-seq pipeline incorporates the assessment of on-target aberrations, providing a broader view of the genotoxicity landscape within the same protocol.^152^ The interrogation of on-target rearrangements is of particular importance, as these are often the most abundant and the most neglected SVs. However, studies have shown that the cell type, the genome editing platform, the locus, or the DNA repair modulators have an impact beyond OTA.^37^^,^^40^^,^^148^^,^^151^^,^^152^^,^^176^ For example, a recent study investigated the differential effects on specificity and SVs when comparing Cas9 nucleases to paired nCas9 in the context of promiscuous gRNAs.^152^ The results showed that the use of a dual nickase approach was valid to mitigate OT effects as previously reported,^177^ but it also altered the profile of on-target aberrations.^152^ This raises an important question: are large on-target aberrations inevitable or will we be able to mitigate their frequency and pattern?

While many OT detection assays were originally optimized for SpCas9, their suitability for other nucleases—particularly those with different DNA cleavage characteristics—remains uncertain. For example, blunt ends generated by Cas9 may be more prone to erroneous rejoining or chromosomal rearrangements than the staggered ends produced by enzymes like Cas12a.^5^ These differences in end structure not only influence the likelihood and nature of SV formation but may also affect how efficiently such events are captured by detection assays. Adapter ligation steps, in particular, may be less effective at capturing staggered ends, potentially leading to underrepresentation of SVs associated with type V nucleases. As the field expands to include a broader array of editors and platforms, there is a growing need to refine or redesign SV detection tools to account for cleavage-specific biases and ensure accurate characterization of genome integrity.

There remains an unmet need for technologies sensitive enough to trace other types of genomic aberrations, such as ultra-low-frequency chromosomal loss or those occurring in complex repetitive sequences. In most cases, SVs and small gene-disrupting edits are detrimental to cell viability, leading to the self-elimination of cells harboring such changes. However, the risk of such events causing malignant cell transformation or affecting tissue-relevant genes cannot be disregarded. We, therefore, call for genotoxicity testing for the detection of SVs to be extended beyond the current state of the art to allow a more comprehensive assessment of the potential spectrum of chromosomal aberrations. Even if we cannot yet predict how such rearrangements will affect patients, we need to have the relevant data today so that we can draw the necessary conclusions in the future if serious side effects occur.

## Genomic stability assessment for genome-editing enhancers

### Enhancing genome-editing precision and efficiency using small molecules

Gene-editing technologies, especially CRISPR-Cas9, have significantly advanced our ability to manipulate genomes with high precision. However, achieving efficient and precise editing remains a challenge, particularly due to OT effects and low rates of HDR, especially in post-mitotic and non-dividing cells. To address the limitations associated with HDR in these contexts, alternative strategies have been developed that harness NHEJ–mediated mechanisms, such as homology-independent targeted integration^178^ and obligate ligation-gated recombination.^179^ While these NHEJ-based methods are effective in achieving targeted insertion, they may not always result in the seamless integration of donor sequences as seen with HDR; instead, they enable efficient cassette incorporation at the desired locus, broadening the range of genomic modifications possible in HDR-refractory tissues.

In addition to these nuclease-based strategies, small molecules have emerged as powerful tools to enhance the efficiency and precision of gene editing.^180^ Recent advancements have improved gene editing outcomes, focusing on enhancing HDR, reducing OT effects, and ensuring genomic stability.^181^^,^^182^ Strategies to improve HDR can be grouped into four main categories: NHEJ inhibition, direct HDR enhancement, reduction of DNA damage sensing, and cell-cycle synchronization. These approaches are under active investigation and hold promise for future clinical applications.

### Cell-cycle synchronization

Most cell-cycle regulatory compounds have shown proof-of-concept activity in immortalized cell lines, with minimal confirmation in target primary human cells such as hematopoietic stem and progenitor cells (HSPCs) or T cells.^183^ Consequently, very few genotoxic studies have been conducted on these compounds. Nocodazole and aphidicolin, which block cells in the M and S phases, respectively, were shown to be relatively cytotoxic to primary cells *in vitro*, limiting their investigative use despite a 10%–30% absolute increase in HDR in immortalized cell lines.^183^ Synchronization close to the G2 phase increases HDR, possibly due to chromatin accessibility, chromosome territories, and nuclear membrane structure instability.

### P53 inhibition

Edited hematopoietic stem cells (HSCs) are significantly affected by culture procedures and the presence of DSBs and DNA donor templates. This is evidenced by increased p21 (G_1_ checkpoint regulator) expression driven by p53 activation and increased 53BP1 loci driven by the detection of open DNA ends.^184^ These factors trigger a signaling cascade that halts cell proliferation. Furthermore, long-term repopulating HSCs characterized by CD34^+^, CD45RA^−^, and CD90^+^ cells show a lower propensity for HDR outcomes. To address these bottlenecks, a medium containing SR-1, UM171, and transient expression of a p53 dominant-negative variant and the adenovirus 5 E4orf6/7 protein has been proposed. The latter upregulates the HDR apparatus in long-term HSCs, resulting in a 50% increase in editing efficiency in long-term human grafts. An additional promising molecule is GSE56, a dominant-negative mutant of the p53 protein. When co-electroporated with the CRISPR-Cas complex, GSE56 has been shown to enhance the engraftment of edited HSCs *in vivo*. These exceptional results were supplemented with genotoxic studies, revealing initial clinical translatability.^185^ However, it is imperative to note that p53 is a crucial tumor suppressor that plays a significant role in regulating the cell cycle, promoting DNA repair, and initiating apoptosis in response to DNA damage.^186^ Inhibition of p53 can have several consequences, particularly in the context of gene editing and HSCs.^187^ While there may be short-term benefits in gene editing, the long-term risks associated with genomic instability and potential transformation into cancerous cells must be considered carefully. The epigenetic modification induced by the E4orf6/7 protein, which dysregulates the activity of the cell-cycle controller E2F, could also be a similar factor of concern. However, the transient nature of these enhancers may bridge the natural cell response to DNA damage for the time needed to edit the cells in a controlled and still safe manner.

### HDR direct enhancement

HDR is a repair pathway shown to be 10–30 times slower than NHEJ in primary cells and cell lines.^167^ To address this gap, a fusion protein bringing the Cas9 nuclease to the cleavage site with CtBP-interacting protein (CtIP) exonuclease and a dominant-negative variant of RNF168 (dnRNF168) has been proposed. This approach recruits HDR factors and inhibits NHEJ initiation.^188^ However, results in primary cells were not confirmed, and chromosomal aberration analysis with CAST-seq exposed an increased number of translocations at the on-target site with one OT site.^188^

### NHEJ and alt-NHEJ inhibition

DSBs can be repaired by different pathways, depending on the targeted loci, cell-cycle state, culture, and cell type. Despite these variations, the NHEJ pathway remains the predominant repair mechanism.^167^ Its inhibition allows time for HDR to exert its activity and recruit the donor cassette at the edited site. Inhibition of DNA-PK, for example using NU7026 or NU7441, has been shown to suppress NHEJ while facilitating a higher frequency of HDR.^178^^,^^189^ STL127705, another inhibitor, targets the Ku70/80 complex.^190^ Inhibition of NHEJ using DNA-PK, coupled with the inhibition of the DNA polymerase theta (PolΘ), which mediated the alt-NHEJ pathway, has proven successful even in primary cells, boosting HDR up to 90% in iPSCs^181^ and HSPCs^191^ from 20% to 40% without drugs. The DNA-PK inhibitor AZD7648 outperformed M3814 in side-by-side studies, showing higher potency and less cytotoxicity. Additionally, the inhibition of 53BP1, a key player in the NHEJ pathway, has been shown to favor HDR, with up to 80% edited alleles utilizing the peptide i53. Deeper characterization showed that large deletions increased during these strategies but were normalized when utilizing the donor template,^192^ making NHEJ inhibition one of the most promising strategies. The inhibition of NHEJ, particularly when targeting canonical NHEJ exclusively, must be approached with caution as it can lead to larger genomic rearrangements via the alt-NHEJ and single-strand annealing pathways.^5^^,^^193^ If NHEJ inhibitors induce significant deletions, then PCR primers designed to detect NHEJ events may no longer anneal effectively. As demonstrated by Cullot et al.,^193^ such large deletions create a misleading impression of reduced NHEJ activity and an apparent increase in HDR events, when in reality, the result simply reflects the presence of large deletions at the target site.

### Detecting genomic instability events induced by small molecules

The incredible efforts to improve HDR are finally yielding significant results in therapeutically relevant cells.^181^^,^^182^ These data are being deeply investigated for genomic instability. For example, DNA-PK inhibition with AZD7648 was shown to increase not only the frequency of large on-target deletions but also the number of induced translocations, both qualitatively and quantitatively.^193^ Recently, a relatively simple approach based on multiplexed digital PCR has been developed that can return a targeted qualification and quantification of editing outcomes.^167^ With this novel tool, it is possible to track the actual effects of NHEJ inhibition, revealing a bias in up to 90% of the alleles mainly due to the difficulty in detecting open ends with other techniques. This work could also demonstrate the “scarless” precise repair, along with insights into recurrent nuclease cutting and DNA repair rates. Furthermore, OTs were shown to have a greater chance of translocating with the on-target site, stressing the importance of accurate screening of designer editors. Despite these promising initial findings, comprehensive and unbiased characterization is still required to fully assess the benefits and potential drawbacks of these strategies and to guide the development of next-generation gene-editing tools. The use of these molecules for *in vivo* gene editing remains a matter of debate, even though some are already clinically approved as chemotherapeutic agents. This caution stems from the fact that many chemotherapies targeting DNA repair pathways are known to increase the risk of secondary malignancies, such as therapy-related myelodysplasia or acute myeloid leukemia. As a result, *ex vivo* gene therapy currently represents the most feasible therapeutic route. Nevertheless, the potential for *in vivo* applications cannot be ruled out, especially as advances in reducing genotoxicity or improving targeted delivery could enhance the safety and efficacy of these approaches for patients.

### Functional consequences of genome-editing repair outcomes

Once an adverse CRISPR-Cas-induced mutation is identified in any of the described methods, it remains to evaluate its potential functional implications. In general, most OT mutations occur within intronic or non-coding regions and are unlikely to impact cellular function.^194^ Even in cases where mutations arise in exonic regions, loss-of-function variants are typically subjected to negative selection and are unlikely to propagate within the patient’s body. Of particular concern, however, are gain-of-function mutations, which, if advantageous, undergo clonal expansion, potentially contributing to tumorigenesis. When considering the mutation genotype, frameshift mutations present a heightened risk of disrupting normal gene function. The presence of large indels and genomic rearrangements is also concerning due to their potential to alter gene expression.

Linking a mutation to its biological effect remains an unresolved challenge. Specific assays can determine whether a specific function of edited cells was disrupted, for example, cytotoxicity, differentiation to subpopulation in progenitors, or infiltration to the target tissue. Computational strategies, such as deep learning algorithms can to some extent predict protein function based on a given mutation,^195^^,^^196^ but they are still limited, especially in non-coding regions. Most of the effort focuses on identifying the oncogenic potential of the engineered cells. State-of-the-art practices track clonal expansion *in vitro* to identify potential advantageous mutations and then sample modified cells over time and monitor specific indels through DNA sequencing or assessing gene expression via RNA-seq.^37^^,^^197^ Additionally, targeted amplification assays like real-time PCR or ddPCR can be employed to quantify specific indels or SVs over time. For a better resolution of linking mutation-phenotype, single-cell sequencing methods can be applied. Some studies incorporated single-cell RNA-seq to measure SVs over time and address the expression profile after editing.^37^^,^^38^ A later technology incorporates targeted single-cell DNA sequencing with immunostaining to achieve a one-to-one characterization of the mutations in an engineered sample to the observed phenotype.^41^

Another strategy for *in vitro* tumorigenicity assessment is using transformation assays, which measure the cell growth as a proxy for oncogenic potential. Soft agar culture assays are commonly used to assess tumorigenicity, wherein cells are seeded on an agar substrate and screened for proliferating clones based on colony size.^198^ Alternatively, the growth in low-attachment assay^199^^,^^200^ measures cell growth by quantifying the ATP production of cells seeded in low-attachment conditions. Both assays have been shown to identify adverse events in CRISPR-Cas-engineered cells with a detection limit of 1%–3%.^201^

The most widely used approach for measuring potential outcomes involves xenotransplantation of edited cells into immunodeficient mice, typically non-obese diabetic severe combined immunodeficiency- γ mice, for a period of weeks to months.^51^^,^^202^^,^^203^^,^^204^ This *in vivo* approach mimics the high cell dosage used in therapeutic products while preserving the “whole organism” context. Xenotransplantation enables the assessment of long-term engraftment, clonal dynamics, and lineage contribution of edited cells in a biologically relevant environment. Importantly, it provides a platform to monitor potential adverse effects, such as transformation or abnormal proliferation, over time, under selective pressures and physiological conditions that cannot be fully replicated *in vitro*. While xenotransplantation remains a powerful tool, it is limited by its extended duration and low throughput, and may lack clear physiological relevance to humans due to species-specific differences in DNA repair mechanisms, immune function, and cellular responses.^205^^,^^206^ Moreover, the relatively small number of engrafted cells and limited sampling sensitivity increases the likelihood of false negative results, making it difficult to detect rare but potentially deleterious mutations. These limitations underscore the need for complementary assays with higher sensitivity and resolution to ensure a more comprehensive evaluation of genome-editing outcomes.

*In vivo* editing presents unique challenges for functional outcome assessment, as the edited cells remain within the organism and cannot be readily monitored or recovered for direct analysis. While deep sequencing can detect specific mutations post-treatment, the limited availability of material often constrains sensitivity. Functional consequences may be assessed using the *in vitro* assays mentioned above; however, their predictive value for *in vivo* outcomes remains constrained. Surrogate approaches, such as editing primary human cells *ex vivo* followed by transplantation into immunodeficient mice, offer partial insights but lack full translational relevance. Although *in vivo* strategies can be evaluated in animal models, including mice and non-human primates, these systems do not fully recapitulate human-specific genomic or physiological contexts. Continued development of improved models and sensitive assays is therefore essential to accurately assess safety and efficacy in the *in vivo* setting.

Monitoring genotoxicity is also important for other factors aside from the CRISPR-Cas cleavage. Viral vector-based HDR templates, for example, are known to trigger the DDR pathway, which triggers p53 activation, and, consequently, apoptosis of modified cells.^184^^,^^207^ *In vivo* studies using recombinant AAV-mediated HDR CRISPR-Cas9 editing demonstrated a reduction in the HDR population over time.^208^ AAV toxicity can partly explain the pancytopenia observed in the first AAV-mediated HDR-based clinical trial aimed at correcting the *HBB* gene, which has since been halted.^209^

Overall, potential risks must be carefully evaluated, and any indications of possible genotoxicity should be flagged, thoroughly assessed, and, where possible, mitigated using the available technologies. While it may not be feasible to entirely eliminate adverse OT effects, it is crucial to monitor and report any potential events to ensure the safe implementation of gene therapy in clinical settings.

### Conclusions

Gene therapy is a promising field with the potential to revolutionize the treatment of many medical conditions. As the risks associated with OTA are well recognized, ongoing advancements—including improved sequencing methods, computational algorithms, and enhanced gRNAs and Cas variants—continue to address these concerns.

Considering the over 100 active clinical trials utilizing genome-editing platforms, robust and comprehensive safety assessment frameworks are essential. Regulatory agencies recommend integrating multiple, orthogonal approaches to reduce bias and improve the reliability of OTA detection. These assessments must go beyond localized edits to include broader evaluations of genomic integrity, encompassing both on- and OT effects. However, in the absence of a universally mandated workflow, the ultimate pipeline remains at the discretion of the product developers, subject to eventual regulatory validation.

Current clinical strategies illustrate this variability. For instance, exa-cel (Casgevy)^210^ and trem-cel^211^ rely on *in silico* prediction combined with GUIDE-seq — their sole empirical assay — to nominate OTA sites. Although no OTA was detected at nominated sites, the impact on SVs remains unclear. In contrast, reni-cel (EDIT-301)^212^ and NTLA-2001^213^ extend their assessment by incorporating *in vitro* nomination assays (Digenome-seq and SITE-seq, respectively), with the latter additionally employing long-read sequencing strategies to detect SVs. These differences in genotoxicity surveys reflect how current frameworks allow for a case-by-case approach shaped by the specific therapeutic strategy and its risk-benefit profile. For example, Casgevy,^210^ designed to inactivate the *BCL11A* erythroid enhancer in autologous HSPCs for β-hemoglobinopathies, was approved despite criticism for relying on a non-representative reference genome. Its *ex vivo* setting, however, enables individualized post-editing OTA profiling before reinfusion into the patient, offering an additional layer of safety. Of note, OTA is often evaluated under a worst-case scenario, such as using high nuclease expression or cell-free *in vitro* assays. In practical settings, such as preclinical or clinical trials conducted under GMP conditions in cellular or tissue contexts, OTA is reported to be significantly lower.^20^^,^^213^^,^^214^ Nevertheless, caution should be taken when interpreting the absence of detectable OTA, as it may reflect limitations in assay sensitivity or the selection of inappropriate detection methods rather than an actual absence of OT effects.

With the progression of available tools and assays, safety assessments should be tailored for each CRISPR-based drug in an experimentally relevant setup, to select optimal gRNA candidates and thoroughly evaluate potential genotoxicity. Given the current limitations of each platform in detecting potential OTs, it is recommended that multiple orthogonal methods , including the *in silico*, cell-free, and cell-based approaches discussed in this paper, be employed to ensure a comprehensive OT assessment. Importantly, many established OT detection assays were developed for SpCas9 and may not be directly applicable to other CRISPR systems or to editors like BE and PE, which do not rely on DSBs. Therefore, adapting or developing new assays that align with the molecular mechanisms of these novel editors will be essential for accurately characterizing their specificity.

To support a more standardized and comprehensive safety evaluation strategy, we propose a five-step framework (Figure 4), encompassing (1) rational gRNA design and optimization, including computational selection, OT minimization, and pairing with engineered Cas variants and delivery strategies suited to the intended application; (2) OT site nomination and validation using *in silico* prediction and orthogonal experimental assays; (3) assessment of SVs and large-scale genomic rearrangements; (4) functional and long-term safety validation in relevant *in vitro* and *in vivo* models; and (5) consideration of regulatory and manufacturing factors, such as GMP compliance and traceability, which, while not the focus of this review, remain essential for clinical translation.

This roadmap is intended to guide developers in selecting the most appropriate tools, assays, and evaluation strategies at each stage of therapeutic development. However, the optimal configuration will inevitably vary depending on the biological system and delivery context. For example, editing HSCs, T cells, or hepatocytes presents distinct challenges in terms of delivery efficiency, genotoxic risk, and assay accessibility. Likewise, *ex vivo* approaches, such as those applied to HSPCs, offer opportunities for individualized, post-editing analysis before reinfusion, while *in vivo* therapies require robust preclinical models to assess biodistribution, immunogenicity, and long-term genotoxicity *in situ*. Therefore, safety assessments must be carefully contextualized to the specific cell type, editing modality, and therapeutic setting, while remaining aligned with rigorous scientific and regulatory standards. Although challenges remain in harmonizing specific standards, thresholds, detection limits, and protocols, the continuous innovation and collaborative efforts in the field offer a path toward achieving a high and consistent level of safety in gene therapy.

## Acknowledgments

This publication is based upon work from COST Action Gene Editing for the treatment of Human Diseases, CA21113 (https://www.genehumdi.eu) supported by 10.13039/501100000921COST (10.13039/501100000921European Cooperation in Science and Technology). J.G. was further supported by the 10.13039/501100009708Novo Nordisk Foundation (NNF21OC0068988) and the Independent Danish Research Foundation (9041-00317B). K.B. was further supported by Consejería de Universidad, Investigación e Innovación under Plan Andaluz de Investigación, Desarrollo e Innovación (PAIDI 2020) (ProyExcel_00875); by the Consejería de Salud y Consumo of the Junta de Andalucía (PI-0216-2024); and by Fundación Mutua Madrileña (FMM-AP16030-2024). K.B. also held a Nicolas Monardes contract from 10.13039/501100023731Consejería de Salud y Consumo, Junta de Andalucía (0006/2018). A.H. and T.C. were also supported by the 10.13039/501100000780European Union under the Horizon Europe grant (101057659 - EDITSCD). T.C. was further supported by the 10.13039/501100001659German Research Foundation (DFG, Project-ID 499552394 – SFB 1597). All figures in this paper were created with BioRender.com.

## Author contributions

N.K.: writing – original draft, writing – review & editing, and visualization; A.S. and V.R.-D.: visualization; G.T., J.G., C.F.-G., K.B., and T.C.: writing – original draft and writing – review & editing; S.R.: writing – review & editing; C.L.: writing – original draft, writing – review & editing, and supervision; A.H.: writing – review & editing and supervision.

## Declaration of interests

A.H. is the founder and chief scientific officer of CassidyBio. CassidyBio did not have input into the design, execution, interpretation, or publication of this work. T.C. and G.T. hold patents on CAST-seq. G.T. is current employees of AstraZeneca and may be AstraZeneca shareholders.

## Declaration of generative AI in scientific writing

During the preparation of this work the authors used available AI tools (e.g., Copilot and ChatGPT) to enhance the quality of our writing. After using these tools, the authors reviewed and edited the content as needed and take full responsibility for the content of the publication.
